# Gitelman syndrome combined with diabetes mellitus: A case report and literature review

**DOI:** 10.1097/MD.0000000000036663

**Published:** 2023-12-15

**Authors:** Xiaoyan Huang, Miaohui Wu, Lunpan Mou, Yaping Zhang, Jianjia Jiang

**Affiliations:** a Department of Endocrinology, Quanzhou First Hospital Affiliated to Fujian Medical University, Quanzhou, China; b School of Pharmacy, Fujian Medical University, Quanzhou, China.

**Keywords:** Diabetes Mellitus treatment, Gitelman syndrome, hypokalemia, metabolic alkalosis

## Abstract

**Rationale:**

Gitelman syndrome (GS) is an uncommon autosomal recessive tubulopathy resulting from a functional deletion mutation in the SLC12A3 gene. Its onset is typically insidious and challenging to discern, and it is characterized by hypokalemia, metabolic alkalosis, and reduced urinary calcium excretion. There is limited literature on the diagnosis and management of GS in individuals with concomitant diabetes.

**Patient concerns:**

A 36-year-old male patient with a longstanding history of diabetes exhibited suboptimal glycemic control. Additionally, he presented with concurrent findings of hypokalemia, hypomagnesemia, hypocalciuria, and metabolic alkalosis.

**Diagnosis:**

Building upon the patient’s clinical manifestations and extensive laboratory evaluations, we conducted thorough genetic testing, leading to the identification of a compound heterozygous mutation within the SLC12A3 gene. This definitive finding confirmed the diagnosis of GS.

**Interventions:**

We have formulated a detailed medication regimen for patients, encompassing personalized selection of hypoglycemic medications and targeted electrolyte supplementation.

**Outcomes:**

Following 1 week of comprehensive therapeutic intervention, the patient’s serum potassium level effectively normalized to 3.79 mmol/L, blood glucose parameters stabilized, and there was significant alleviation of clinical symptoms.

**Lessons:**

GS has a hidden onset and requires early diagnosis and intervention based on patient related symptoms and laboratory indicators in clinical practice, and personalized medication plans need to be provided according to the specific situation of the patient.

## 1. Introduction

Gitelman Syndrome (GS) represents an autosomal recessive hereditary renal disorder characterized by hypokalemia, hypomagnesemia, hypocalciuria, and metabolic alkalosis. This disorder is featured in China’s First Catalog of Rare Diseases.^[1]^ A comprehensive review of both domestic and international literature reveals that while substantial genetic analyses of GS have been conducted since its genetic analysis in 1996, there remains a need for further exploration and research into its clinical diagnosis, treatment, and follow-up.^[2]^ This paper presents a comprehensive summary and discussion of a case where GS is combined with diabetes mellitus and has been admitted to our department, drawing insights from pertinent literature.

## 2. Case presentation

A 36-year-old male patient presented with a chief complaint of chronic hyperglycemia, accompanied by limb paresthesia persisting for 1 month. The patient received a diagnosis of diabetes mellitus 14 years prior and was managed with oral hypoglycemic agents. Fasting blood glucose levels demonstrated periodic fluctuations ranging between 7 to 9 mmol/L. A longstanding history of hypokalemia emerged 15 years earlier during a routine medical assessment, evidenced by fasting serum potassium levels oscillating around 2.5 mmol/L. The patient has reported intermittent numbness in the hands and feet, with an absence of accompanying symptoms such as nausea, vomiting, abdominal distension, chest tightness, or palpitations. Previously diagnosed with Bartter syndrome in an external hospital. The patient intermittently self-administered potassium citrate (2 sachets Tid), yielding post-treatment serum potassium levels of approximately 4.0 mmol/L. There existed no antecedent diagnoses of hypertension or chronic diarrhea. The patient’s reproductive history indicated a marital status with 2 healthy children. Familial antecedents disclosed that 1 sister had been diagnosed 15 years ago with a co-occurrence of diabetes mellitus and Bartter syndrome, while both the maternal mother and grandmother had a history of diabetes mellitus.

### 2.1. Physical examination

Vital signs revealed a body temperature of 36.0°C, a heart rate of 83 beats/min, a respiratory rate of 19 breaths/min, and blood pressure of 101/65 mm Hg. The patient’s BMI was calculated at 19.27 kg/m^2^. Developmental progress was within normal parameters. Mental acuity was intact. Pulmonary auscultation indicated unobstructed respiratory sounds bilaterally, while cardiac auscultation revealed robust and rhythmic heart sounds. The abdomen exhibited a flat and supple contour without any evidence of peripheral edema. Notably, the patient displayed reduced sensory perception for pain, touch, and temperature in both lower extremities, as evidenced by a positive response to the 10 g nylon filament test. Muscle strength assessment revealed grade V in all limbs.

### 2.2. Ancillary tests

Hematological analyses disclosed the subsequent outcomes: a white blood cell count of 8.41 × 10^9/L, a neutrophil percentage (NE%) of 65.6%, a red blood cell count of 6.21 × 10^12/L, a hemoglobin level of 191 g/L, a hematocrit of 53.5%, and a platelet count of 232 × 10^9/L. Erythropoietin level manifested at 5.9 IU/L, and the Breakpoint Cluster Region-Abelson Leukemia Virus fusion gene test yielded negative results. Similarly, the jAK2 exon 14 mutations also returned negative findings. Morphologic assessment of a bone marrow smear corroborated the presence of erythrocytosis. Sonographic imaging of the urinary tract unveiled multifocal cystic formations within both kidneys, along with numerous calcified foci embedded within the renal parenchyma. Further pertinent examinations are presented in Table 1.

**Table 1:** Clinical examination findings in patients.

Test items	Specific items	Measured values	Normal values
Islet-related function	Fasting serum C-peptide (µg/L)	2.45	1.1–4.4
	2 h serum C-peptide (µg/L)	6.38↑	1.34–2.50
	Glycosylated hemoglobin (%)	8.4↑	4–6
	Anti-Islet cell antibodies	Negatives	Negatives
	Anti-Insulin antibodies	Negatives	Negatives
	Anti-glutamic acid decarboxylase antibody	Negatives	Negatives
Blood biochemistry	Potassium (mmol/L)	3.15↓	3.5–5.3
	Sodium (mmol/L)	137	137–147
	Chloride (mmol/L)	96↓	99–110
	Calcium (mmol/L)	2.55↑	2.11–2.52
	Magnesium (mmol/L)	0.61↓	0.75–1.02
	Carbon dioxide binding capacity (mmol/L)	29	22–29
	Urea (mmol/L)	6.64	3.1–8.0
	Creatinine (µmol/L)	78.6	53–97
24-hour urine sample biochemical tests	potassium (mmol/L)	84.6	25–100
	Calcium (mmol/L)	2.0↓	2.5–7.5
	Sodium (mmol/L)	228	130–260
	Phosphorus (mmol/L)	22.59↓	32.3–38.4
	Urine output (L/24 h)	3.0↑	0.5–2.8
Blood gas analysis	Lactate (mmol/L)	2.0↑	0.5–1.6
	pH	7.485↑	7.35–7.45
	PaCO2 (mm Hg)	41.7	35–45
	cBase (ecf)	8.0↑	−3 to 3
	HCO3^-^ (mmol/L)	31.0↑	22–26
RAAS (standing)	Aldosterone (pg/mL)	422.1↑	40–310
	Renin (pg/mL)	90.9↑	4–38
	Angiotensin (pg/mL)	187.2↑	10–88
	Aldosterone/renin concentration ratio	4.64	≤32

**Figure 1.**
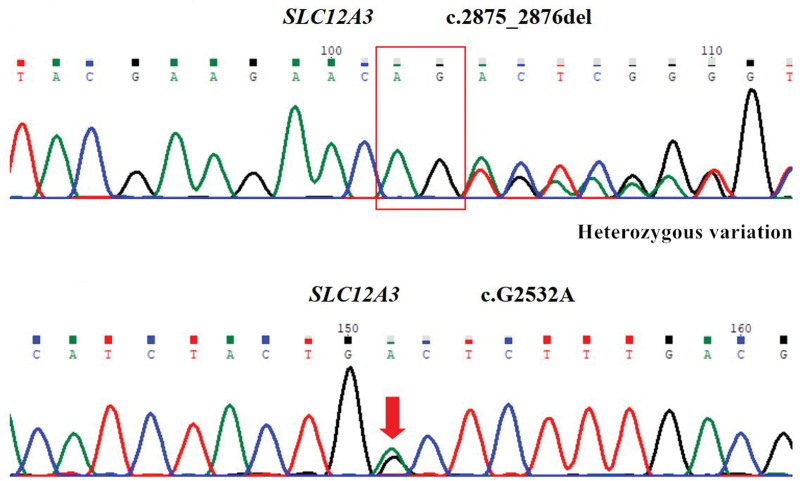
The sequencing peak map of the patient is displayed. A targeted screening was conducted to identify specific genetic variations associated with GS syndrome within the SLC12A3 gene. This screening revealed the presence of the c.2875_2876del and c.G2532A variant sites, which exhibit a strong correlation with the patient’s clinical phenotype. As a result, the patient was diagnosed with a compound heterozygous pathogenic variant.

After admission, the patient received oral potassium chloride solution at a dose of 10 mL 3 times a day on the same day. In addition, the patient was prescribed spironolactone at a dose of 20 mg 3 times a day. After 2 days of treatment, serum potassium levels were successfully restored to 3.79 mmol/L. Concurrently, a medication regimen comprising insulin glargine, gliclazide sustained-release tablets, acarbose, and sitagliptin was instituted to achieve glycemic control. After a week of observation and treatment, blood glucose parameters stabilized. On the 8th day of admission, with the patient and their family’s consent, peripheral blood samples were collected and sent to the Da’an Clinical Testing Center in Guangzhou, China, for comprehensive gene sequencing. The results of the genetic analysis are presented in Figure 1 and Table 2.

**Table 2:** Patient-Associated genetic testing data.

Gene	Genomic locus	Allelic variation details	Zygosity type	Hereditary pattern	Pathogenicity grade
HNF1B	Chr17:36104769	NM_000458:c. C107T:p.S36F	Heterozygous	AD	Clinical significance unknown
SLC12A3	Chr16:56936412	NM_000339:c.2875_2876del.p.R959Sfs*11	Heterozygous	AR	Potential Pathogenicity
SLC12A3	Chr16:56926950	NM_000339:c. G2532A.p.W844X	Heterozygous	AR	Pathogenic

AD = autosomal dominant, AR = autosomal recessive.

Utilizing the comprehensive dataset and pertinent outcomes of genetic analyses, the ultimate diagnosis was delineated as follows: Diabetes Mellitus (awaiting precise subtype characterization), concurrent with Diabetic Nephropathy (Stage G1A2) and Diabetic Peripheral Neuropathy; Gitelman Syndrome; Erythrocytosis; and Mixed Hyperlipidemia.

These diagnostic attributions epitomize the amalgamation of genetic insights and clinical manifestations, underscoring the intricate intertwining of multiple morbidities within this patient. This intricate diagnostic landscape holds paramount clinical significance, warranting comprehensive and insightful discussions.

## 3. Discussion

### 3.1. Diagnosis of GS

GS typically onsets during adolescence or adulthood, characterized by clinical features primarily centered around hypokalemia, hypomagnesemia, and renin-angiotensin-aldosterone system (RAAS) activation. These manifestations frequently encompass nonspecific symptoms such as fatigue, xerostomia, polyuria, muscle weakness, spasms, tetany, and hypokalemia. Notably, certain individuals may remain asymptomatic, often leading to misdiagnoses as sporadic hypokalemia or hypomagnesemia.^[3]^ Nonetheless, Severe adverse events have also been reported in the literature, encompassing growth retardation,^[2,4]^ chondrocalcinosis,^[5]^ generalized seizures,^[6,7]^ non-periodic paralysis,^[2]^ rhabdomyolysis,^[8]^ and even cardiac arrest.^[9]^ The course of GS typically follows a mild trajectory, characterized by predominantly modest clinical symptoms. However, delayed medical attention often ensues due to symptom underestimation, potentially impeding timely diagnosis and intervention. Therefore, early identification, diagnosis, and intervention play a pivotal role in enhancing patient prognosis and quality of life.^[10]^

As the clinical manifestations of GS lack specificity, laboratory investigations often serve as the frontline indicator for diagnosing this condition. Typical laboratory findings manifest as the “five lows and one high” pattern, accompanied by metabolic alkalosis. Specifically, this pattern entails low blood potassium, low blood magnesium, low blood chloride, low urinary calcium, mildly reduced blood pressure, and elevated RAAS activity.^[11]^ Additionally, GS patients may also present with insulin resistance. literature reports also have documented the occurrence of uncommon hypophosphatemia and hyponatremia.^[12]^

Upon examination, the patient in this case was found to exhibit chronic renal potassium wasting, low blood magnesium and chloride levels, normal blood calcium but reduced urinary calcium levels, mildly reduced blood pressure, elevated levels of renin and aldosterone prior to treatment, and a normal aldosterone-to-renin ratio. Considering that severe vomiting and excessive use of Liquorice, diuretics, and laxatives can also lead to similar symptoms, a comprehensive review of the clinical history and medication usage is imperative. If necessary, diuretic screening and urinary chloride excretion measurements should be undertaken.^[13]^ Following the exclusion of these factors, the likelihood of GS becomes more prominent.

As a genetically-associated syndrome, GS diagnosis necessitates genetic testing. GS is primarily linked to inactivating mutations on the SLC12A3 gene located on chromosome 16q13. This gene encodes the thiazide-sensitive sodium-chloride cotransporter (NCCT), a protein situated on the apical membrane of the distal convoluted tubule in the kidney.^[14]^ Functional loss mutations within SLC12A3 lead to structural and/or functional anomalies of NCCT, disrupting sodium-chloride reabsorption in the distal convoluted tubule,^[15]^ subsequently triggering a series of pathophysiological and clinical manifestations, including hypovolemia, RAAS activation, hypokalemia, and metabolic alkalosis. Recent studies have also highlighted similar symptoms in patients with mutations in CLCKNB, KCNJ10, FXYD2, and HNF1B genes,^[3,13]^ emphasizing the necessity for comprehensive consideration during GS diagnosis. In this case, the patient’s final diagnosis of GS was confirmed through genetic testing, revealing heterozygous mutations on the SLC12A3 gene locus.

### 3.2. Identification of GS with similar diseases

Bartter Syndrome, GS, Liddle Syndrome, and licorice toxicity all have the potential to induce hypokalemia and metabolic alkalosis.^[16]^ However, Liddle Syndrome and licorice toxicity are often distinguished by hypertension and aldosterone suppression, characteristics that set them apart from Bartter syndrome (BS) and GS. While both BS and GS share analogous clinical manifestations and biochemical features including hypokalemia, nephrogenic potassium loss, hypochloremic metabolic alkalosis, activation of the renin-angiotensin-aldosterone system (RAAS), and normal or low blood pressure, clinical differentiation remains a relatively intricate endeavor.

GS was historically viewed as a subtype of BS. Nevertheless, with the successful cloning of the GS-causing gene, it is now acknowledged as an independent disorder. Both BS and GS represent autosomal recessive salt-depletion disorders featuring secondary aldosteronism and (typically) hypotension.^[17]^ Despite their parallels, they differ in terms of molecular underpinnings and clinical presentations. Clinical differentiation can be achieved through considerations such as age of onset, serum and urine biochemical markers, and physiological function tests.

GS is often incidentally discovered upon routine blood tests detecting hypokalemia. A distinctive hallmark of GS is hypocalciuria, potentially associated with ectopic calcification. Conversely, increased urinary magnesium excretion can contribute to hypomagnesemia. The salt-wasting phenotype of BS, in contrast, tends to be more severe than that of GS, with diagnosis frequently established during infancy or early childhood.^[17]^ BS is frequently marked by hypercalciuria, potentially leading to renal stone formation or renal calcification. Notably, urine and serum magnesium levels in BS patients are generally normal or low.^[16]^ Nonetheless, when distinguishing between BS and GS, a subset of patients with BS might not exhibit low serum magnesium levels. Preliminary differentiation can be attempted through the urinary calcium excretion rate index (urinary calcium/creatinine ratio). For practical considerations, the chloride clearance test (a clinical physiological function test) utilizing hydrochlorothiazide and furosemide can also be employed. This convenient and cost-effective test is well-suited for differentiating between GS and BS across healthcare settings.^[1]^

Unlike GS, which originates from inactivating mutations in the SLC12A3 gene, BS primarily stems from 5 known gene defects: SLC12A1, KCNJ1, CLCNKB, BSND/CLCNKA/CLCNKB, and CaSR.^[18–20]^ Accurate identification of these genetic defects is paramount for diagnosis. Furthermore, discerning distinct phenotypes during diagnosis provides essential clinical guidance for targeted therapies.

### 3.3. GS combined with diabetes

In this case, the patient exhibited elevated blood glucose levels during youth, without a tendency towards ketosis, and did not rely on insulin hypoglycemic therapy at disease onset. With a history of diabetes mellitus in his grandmother, mother, and sister, maternal inheritance, specifically Maturity-Onset Diabetes of the Young (MODY), was considered and investigated through genetic testing. However, the results did not support a MODY diagnosis. Literature review indicated that 14% to 60% of GS patients may have abnormal glucose metabolism,^[21]^ and the prevalence of diabetes is higher in GS patients compared to the general adult population.^[21–23]^ The primary contributors to diabetes in GS patients are considered to be hypokalemia and hypomagnesemia.^[22]^ Hypokalemia hampers the closure of ATP-sensitive potassium channels and L-type calcium channels on β-cell surfaces, impeding glucose-stimulated insulin secretion.^[24–27]^ Magnesium plays a pivotal role in over 300 enzymatic reactions, particularly phosphorylation. Hypomagnesemia has been shown to diminish tyrosine kinase activity at the insulin receptor level, resulting in decreased insulin-receptor interactions.^[22,28,29]^ Additionally, RAAS activation may lead to insulin resistance.^[30]^ Despite these insights, the precise mechanism of abnormal glucose metabolism and insulin secretion in GS patients remains elusive and warrants further exploration in clinical trials.

In this case, the patient’s irregular follow-up for treatment and the manifestations of low blood potassium, magnesium, and RAAS activation likely contributed to poor glycemic control. Thus, regular monitoring of blood glucose and electrolytes, coupled with timely magnesium and potassium supplementation, as well as appropriate treatment with drugs like spironolactone, may lead to some improvement in impaired glucose metabolism.^[25]^ After receiving symptomatic treatment, the patient displayed significant enhancement in glycemic control, further reinforcing the strong connection between GS and diabetes.

The selection of hypoglycemic agents for patients with GS necessitates a personalized approach, taking individual circumstances into account. In the majority of GS patients, there is a decline in pancreatic insulin secretion.^[12,23]^ Insulin secretagogues, such as sulfonylureas or gliptins, are viable options in these cases. This rationale underscored our decision to employ gliclazide, In the context of utilizing such medications, maintaining meticulous blood glucose surveillance is vital to avert hypoglycemia. α-glucosidase inhibitors, by retarding postprandial carbohydrate absorption, mitigate postprandial hyperglycemia with a lesser risk of hypoglycemia. Hence, we have opted for acarbose as one of the medications within our therapeutic regimen. DPP-4 inhibitors and GLP-1 receptor agonists stimulate insulin release in a glucose-dependent manner while restraining glucagon secretion. Considering the patient’s lower BMI (19.27 kg/m²) and the minor impact of DPP-4i on weight, coupled with its oral convenience and favorable tolerability,^[31]^ we incorporated sigliptin into the therapeutic regimen.

Studies indicate that aldosterone excess can lead to insulin resistance.^[32–34]^ Although metformin is clinically employed to address insulin resistance, given the patient’s concurrent diabetic nephropathy and elevated lactate levels (2 mmol/L), Considering the potential risk of lactic acidosis,^[35]^ the inclusion of metformin in this treatment has been withheld. SGLT-2 inhibitors lower blood glucose by inhibiting renal proximal tubular reabsorption of glucose and sodium, potentially exacerbating the patient’s hypotensive symptoms (101/65 mm Hg). Consequently, this class of medication was excluded from the treatment plan.

It is worth noting that when managing GS patients, in cases of markedly elevated blood glucose levels accompanied by pronounced hyperglycemic symptoms or inadequate glycemic control despite oral hypoglycemic treatment, combined therapy with insulin and oral antidiabetic agents can be considered. However, caution must be exercised to avoid high-dose insulin administration. Insulin use when blood potassium levels fall below 3.3 mmol/L may elicit severe arrhythmias and respiratory muscle weakness.^[36]^

### 3.4. Reflections on the GS diagnosis and treatment process

The levels of serum or urine electrolytes are easily influenced by intake of NaCl, K+, Ca2+, Mg2+, and other factors. In this case, patient’s 24-hour urine electrolyte measurements were synchronized with concurrent blood and urine electrolyte measurements to minimize testing errors. However, in clinical diagnostic practice, it is advisable to conduct 2 to 3 synchronized blood and urine electrolyte measurements to ensure the accuracy of results. Additionally, it is important to discontinue relevant medications (such as antibiotics, diuretics, antineoplastic drugs, etc.) 48 hours before laboratory testing.

On the other hand, for outpatient screening and follow-up of non-admitted patients, collecting 24-hour urine samples can be challenging and inconvenient. In such cases, clinicians can calculate electrolyte excretion fractions or use the ratio of urine electrolytes to urine creatinine for initial assessment. Chloride ion clearance tests can also be employed for determination, as these indices offer better stability and higher sensitivity compared to single-time blood and urine electrolyte measurements.

GS currently lacks a cure, necessitating lifelong magnesium and potassium supplementation through dietary or pharmacological means. The optimal targets for control are serum potassium at 3.0 mmol/L and serum magnesium at 0.6 mmol/L.^[37]^ Given that magnesium supplementation can reduce urinary potassium excretion,^[38]^ generally, magnesium levels in GS patients should be the first concern. In this case, the patient’s blood magnesium level was > 0.6 mmol/L and, combined with the absence of apparent symptoms, magnesium supplementation was not deemed necessary. Reports indicate that some individuals with initially normal magnesium levels may progress to hypomagnesemia.^[39]^ In such cases, magnesium chloride can be considered for supplementation. While magnesium salts like magnesium citrate and magnesium lactate have superior bioavailability, magnesium chloride also compensates for urinary chloride ion loss.^[13]^

Regarding the patient’s hypokalemic status, options include potassium chloride or extended-release potassium citrate tablets. Severe hypokalemia or hypomagnesemia necessitates treatment through intravenous fluids. Aldosterone excess, a characteristic of GS, stimulates urinary potassium excretion, exacerbating hypokalemia. Spironolactone, as a mineralocorticoid receptor antagonist, counteracts aldosterone’s effects, increasing serum potassium concentration. Research suggests that the combined use of aldosterone antagonists (such as spironolactone, eplerenone) and potassium supplements yields superior outcomes compared to using these agents individually.^[40]^ So far, no studies have directly compared spironolactone and eplerenone in terms of efficacy. Spironolactone’s anti-androgen activity can lead to side effects like gynecomastia, while eplerenone, a selective aldosterone receptor antagonist, has negligible affinity for androgen and progesterone receptors, making it a more advantageous option.^[1]^ Further research demonstrates that ACE inhibitors, ARBs, and cyclooxygenase inhibitors can also be employed in GS treatment. These medications rapidly elevate serum potassium levels, suppress renin and aldosterone activity, and are suitable for cases where patients are intolerant to large electrolyte supplementation. They can also improve refractory hypokalemia and hypomagnesemia.^[41–43]^

Importantly, gender can impact prognosis. Some studies indicate that male GS patients tend to experience more severe symptoms and poorer treatment outcomes^.[44,45]^ High doses of electrolyte supplementation might result in side effects like gastric ulcers, vomiting, diarrhea, and electrolyte imbalances.^[37]^ Therefore, it is essential to be mindful of the potential adverse reactions associated with the long-term use of the aforementioned medications. A careful evaluation of the pros and cons should precede the selection of medications. Moreover, patients should be advised to take electrolyte supplements in divided doses during meals, as this can help alleviate gastrointestinal-related symptoms. In this case, the patient’s sister exhibited similar clinical manifestations. While we strongly recommended genetic testing with the patient’s sister, unfortunately, this was declined.

In subsequent treatment, we will actively encourage genetic sequencing within the affected family to ascertain the precise mechanism of mutation and the disease subtype. This approach will facilitate the development of individualized treatment strategies and prognosis estimation. Furthermore, interventions for carriers without symptoms within the family, including early lifestyle modifications and regular follow-up monitoring, could potentially delay or even reverse disease progression.

Through clinical presentation, laboratory test results, and SLC12A3 gene testing, the patient in this case was definitively diagnosed with GS. However, the renal ultrasound showed multiple calcifications in both kidneys, which contradicted the typical GS presentation. Coupled with the low blood magnesium feature, the possibility of familial hypomagnesemia with hypercalciuria and nephrocalcinosis cannot be entirely ruled out. The extent to which renal dysfunction can be attributed solely to diabetes remains to be explored. Further CLDN16 gene testing and renal biopsy could aid in differentiation. Research underscores the close association between GS and diabetic nephropathy, especially with the significant genetic impact of the SLC12A3 gene on renal diseases.^[46]^ Additionally, the patient in this case has concurrent GS, diabetes, and polycythemia. After conducting an extensive literature review, we found no studies that discuss a connection between polycythemia and GS. Given GS’s clear genetic mutation basis, future investigations could delve into experimental exploration from a genetic perspective with larger sample sizes.

## 4. Conclusion

As a specialized physician, it’s crucial to enhance understanding of GS, improve disease detection sensitivity, and strive for accurate diagnosis and treatment. When GS patients are afflicted with other conditions, adhering to personalized treatment principles is essential to maximize their quality of life.

## Author contributions

**Conceptualization:** Lunpan Mou, Jianjia Jiang.

**Methodology:** Lunpan Mou, Yaping Zhang.

**Resources:** Xiaoyan Huang, Miaohui WU, Lunpan Mou, Yaping Zhang.

**Validation:** Jianjia Jiang.

**Writing – original draft:** Xiaoyan Huang.

**Writing – review & editing:** Xiaoyan Huang, Miaohui WU, Yaping Zhang, Jianjia Jiang.
